# Sub-chronic toxicity of polystyrene microplastics in *Danio rerio*: evidence from hematological, biochemical, oxidative stress, and histopathological biomarkers

**DOI:** 10.3389/ftox.2026.1960709

**Published:** 2026-10-20

**Authors:** Palanisamy Devapriya, Suresh Kalaivani, Velliangiri Devi, Ramakrishnan Poopal, Rajan Maheswaran

**Affiliations:** 1 Entomology Laboratory, Department of Zoology, School of Life Sciences, Periyar University, Salem, Tamil Nadu, India; 2 Department of Environmental Science, Periyar University, Salem, Tamil Nadu, India

**Keywords:** *Danio rerio* (zebra fish), histopathological alterations, multi-biomarker assessment, neurophysiological response, polystyrene microplastics, sub-chronic exposure

## Abstract

Polystyrene microplastics (PS-MPs) have emerged as widespread aquatic contaminants with the potential to induce multiple toxic effects in fish. This study investigated the physicochemical characteristics and sub-chronic toxicity of PS-MPs in *Danio rerio* over 20 days. SEM revealed irregular and aggregated PS-MPs with micrometre-scale dimensions (approximately 1–200 µm), while DLS showed a hydrodynamic diameter of 1,115.5 nm with a polydispersity index (PDI) of 0.569. Acute toxicity analysis determined the LC_50_ value of PS-MPs to be 89.1 μg/L, and morphogenetic abnormalities were also observed. Based on the LC_50_, the fish were exposed to Treatment I-2.5 μg/L, Treatment II-25 μg/L, and an untreated control; the sub-chronic exposure induced noticeable physiological, biochemical, and histopathological alterations. Hematological analysis revealed a significant increase in RBC count and a decrease in WBC count in exposed fish. Plasma glucose and total protein levels were significantly reduced, indicating metabolic impairment. LPO levels increased in the gills, demonstrating enhanced oxidative damage. Antioxidant responses were tissue-specific, with SOD and GPx activities significantly elevated in the gill, gut, and brain, whereas GST activity decreased in all three tissues. AChE activity was markedly reduced in the brain, indicating an altered neurophysiological response following PS-MP exposure. Histopathological examination revealed alterations in the gill, gut, and brain tissues of exposed fish. Overall, these findings demonstrate that prolonged exposure to PS-MPs induces oxidative stress, metabolic disturbances, neurotoxicity, immune dysregulation, and tissue damage in *D. rerio*, highlighting the ecological risks posed by microplastic pollution in freshwater ecosystems.

## 1 Introduction

The exponential growth in global plastic production over the past decades has resulted in the widespread accumulation of plastic debris in terrestrial and aquatic ecosystems. Plastics are synthetic polymeric macromolecules such as polyethylene (PE), polypropylene (PP), polyvinyl chloride (PVC), polyethylene terephthalate (PET), and polystyrene (PS), which are valued for their durability, lightweight nature, and versatility. Although plastics provide substantial societal benefits in packaging, construction, transportation, and healthcare, their persistence and resistance to degradation have led to serious environmental concerns (Iroegbu et al., 2021; Napper and Thompson, 2023). Unlike natural polymers such as cellulose and natural rubber, synthetic plastics are recalcitrant and can persist in the environment for decades to centuries.

Plastics are broadly categorized into thermoplastics and thermosets. Thermoplastics (e.g., PE, PP, PVC, PET, and PS) soften upon heating and can be remolded, accounting for the majority of global plastic consumption (Alhazmi et al., 2021). Their widespread use, combined with inefficient waste management, contributes to environmental leakage during both usage and end-of-life stages (Horton, 2022). Once discarded, plastic waste undergoes fragmentation through physical, chemical, and biological processes, resulting in microplastics (MPs), defined as particles smaller than 5 mm (Arthur et al., 2009). Microplastics are classified as primary (intentionally manufactured in micro-sized form for use in cosmetics, personal care products, and industrial abrasives) or secondary (formed by the breakdown of larger plastic debris) (Yu et al., 2020). Secondary microplastics constitute the majority of environmental MPs due to extensive macroplastic pollution (Efimova et al., 2018). These particles exhibit diverse morphologies, sizes, colors, and polymer compositions, and can adsorb heavy metals and persistent organic pollutants, thereby acting as vectors for toxic substances (Rochman et al., 2019; Kooi et al., 2021).

Among various polymers, PS (C_8_H_8_)_n_ is of particular concern due to its extensive industrial and commercial applications. PS is an aromatic thermoplastic polymer synthesized from styrene monomers derived from ethylene and benzene. It is widely used in food packaging, disposable utensils, laboratory ware, insulation materials, and consumer products (Hwang et al., 2020). Its low density and lightweight properties hinder efficient recycling, resulting in substantial environmental accumulation. Once in aquatic systems, PS-MPs and nanoplastics (PS-NPs) may aggregate in saline conditions and interact with biotic and abiotic components, posing risks to aquatic organisms (Hwang et al., 2020).

Accumulating evidence indicates that PS-MPs induce oxidative stress, inflammation, metabolic disruption, neurotoxicity, and transgenerational effects in aquatic organisms (Siddiqui et al., 2023; Nugnes et al., 2022). Mechanistically, PS-MPs can generate reactive oxygen species (ROS), leading to lipid peroxidation (LPO), DNA damage, and impairment of antioxidant defense systems. Studies have reported upregulation of pro-inflammatory genes (e.g., *il-1β*) and activation of NF-κB signaling pathways following PS-MP exposure (Yang et al., 2022). Furthermore, PS-MPs have been shown to suppress key antioxidant enzymes, including superoxide dismutase (SOD), glutathione S-transferase (GST), and glutathione peroxidase (GPx), thereby disrupting cellular redox homeostasis (Hayes et al., 2005; Han et al., 2022). Neurotoxicity has also been associated with altered acetylcholinesterase (AChE) activity, indicating disruption of cholinergic signaling (Colovic et al., 2013).

The zebrafish (*D. rerio*) is a well-established vertebrate model widely employed in genetics, developmental biology, and environmental toxicology. Its small size, rapid reproduction, transparent embryos, and high genetic homology with humans make it particularly suitable for mechanistic toxicological investigations (Howe et al., 2013). Previous studies have demonstrated that exposure to MPs of various polymers causes intestinal damage, oxidative stress, behavioral alterations, and immune dysregulation in zebrafish (Lei et al., 2018). However, comprehensive evaluations integrating enzymatic antioxidants, non-enzymatic antioxidant status, neurotoxicity biomarkers, and histopathological alterations under sublethal PS-MPs exposure remain limited. Therefore, the present study aimed to investigate the sublethal toxicity of PS-MPs in adult *D*. *rerio* following exposure to low and high concentrations derived from the 96-h LC_50_ value. Specifically, this study evaluated hematological and plasma analysis, oxidative stress biomarkers (SOD, GST, GPx, LPO), acetylcholinesterase activity, and histopathological changes in vital organs.

Unlike previous investigations that primarily focused on single endpoints or larval stages, this study provides an integrated multi-biomarker assessment in adult zebrafish under environmentally relevant sublethal concentrations of PS-MPs. By correlating antioxidant defense responses, neurotoxicity markers, and tissue-level damage, the research offers mechanistic insight into polymer-specific toxicity patterns of PS-MPs. This integrative approach enhances understanding of PS-MPs induced oxidative and neurochemical disruptions and contributes to ecological risk assessment frameworks for microplastic pollution in freshwater ecosystems.

## 2 Materials and methods

### 2.1 Experimental animal

Healthy *D. rerio* (AB strain, mean weight: 0.6 ± 0.4 g, mean length: 3.4 ± 0.4 cm) was procured from the local supplier, Shiv Aquarium, located in Salem, Tamil Nadu, India. The specimens were acclimatized for a duration of 14 days under controlled laboratory conditions with continuous aeration provided. Dechlorinated tap water was utilized consistently throughout the course of the present investigation. The parameters of water quality, including temperature (28.4 °C ± 1.20 °C), pH (6.98 ± 0.06), and dissolved oxygen (6.4 ± 0.4 mg/L) (APHA, 2005), were meticulously regulated throughout the entire study period. The photoperiod implemented for the experiment was a 12-h light and 12-h dark cycle. During the acclimatization phase, the fish were provisioned with commercially available pellets containing 28% protein, administered bidaily (morning and evening). Daily, uneaten pellets and metabolic byproducts were systematically removed by manual means. The fish that underwent acclimatization served as the stock for subsequent experiments.

### 2.2 Chemicals required

PS-MPs (C_8_H_8_)_n_ (For synthesis 194345, CAS-No: 9003-53-6, Melt flow index (200 °C/5 kg), 2.5–3.5 g/10 min) were purchased from Central Drug House, India. Other laboratory chemicals used in the present study were purchased from HiMedia, India.

### 2.3 Characterization of PS-MPs

The morphology of PS-MPs was examined using scanning electron microscopy (SEM; Hitachi S-4000) operated at 20 kV. Crystallinity was determined by X-ray diffraction (XRD; Rigaku Smart-Lab SE) over a 2θ range of 10°–80° at 40 kV and 30 mA. Functional groups were identified using Fourier transform infrared spectroscopy (FTIR; PerkinElmer) in the range of 4,000–400 cm^-1^ employing the KBr pellet method. Raman spectra were recorded using a confocal Raman microscope equipped with a 532 nm laser over a spectral range of 400–3,200 cm^-1^. The hydrodynamic particle size and polydispersity index (PDI) were measured by dynamic light scattering (DLS) after sonication of PS-MPs in double-distilled water. A 1 mg/mL PS-MP stock suspension was prepared in double-distilled water, continuously stirred and sonicated to ensure uniform dispersion, and diluted with the respective exposure medium to obtain the desired test concentrations. The Physicochemical characterization results of PS-MPs are shown in Table 1.

**TABLE 1:** Physicochemical characterization of PS-MPs.

Characterization parameter	Technique	Results
Polymer	-	Polystyrene (PS)
Surface morphology	SEM	Irregular, fragmented and aggregated structures with rough, frayed and jagged surfaces
Observed particle dimensions	SEM	Approximately 1-200 µm
Hydrodynamic diameter	DLS	1115.5 nm
Polydispersity index	DLS	0.569
Aggregation Characteristics	DLS/SEM	Heterogeneous particle population with aggregation in aqueous suspension.
Crystalline/structural characteristics	XRD	Reflections at approximately 9.50^0^, 18.5^0^, 38.2^0^, 43.9^0^, 64.4^0,^ and 77.6^0^ (2θ)
Raman bands	Raman spectroscopy	621 cm^-1^, 792 cm^-1^, 1001 cm^-1^, 1194 cm^-1^, 1326 cm^-1^, 1448 cm^-1^, 1600 cm^-1^, 2909 cm^-1^, 3059 cm^-1^
FTIR bands	FTIR	3296.63 cm^-1^, 2921.27 cm^-1^, 2851.91 cm^-1^, 1742.15 cm^-1^, 1540.19 cm^-1^, 1466.75 cm^-1^, 1242.35 cm^-1^, 1170.95 cm^-1^, 697.67 cm^-1^ and 540.59 cm^-1^

### 2.4 Acute toxicity test

Acute toxicity of PS-MPs was evaluated following the OECD Test Guideline 203 under static exposure conditions. Groups of ten *D. rerio* were exposed to PS-MPs at concentrations of 50, 100, 150, 200, and 250 μg/L for 96 h, with a control group maintained under identical conditions. Each treatment was performed in triplicate, and fish were not fed during exposure. Mortality and morphogenetic abnormalities were recorded at regular intervals, and dead fish were removed immediately. The 96-h LC_50_ value and its 95% confidence limits were calculated using the probit analysis methods described by Bliss (1935) and Sokal and Rohlf (2013).

### 2.5 Sub-chronic toxicity study

The exposure concentrations were selected based on the experimentally determined 96-h LC_50_. A semi-static test was conducted to investigate the sub-chronic toxic effects of PS-MPs. *Danio rerio* (10 individuals per set) were exposed to Treatment I (2.5 μg/L) and Treatment II (25 μg/L) for 20 days, with an unexposed control maintained under identical conditions. Each group was maintained in triplicate. Before exposure, feeding was withheld for 24 h. During the exposure period, fish were fed *ad libitum* with commercially available feed twice daily. Uneaten feed and fecal matter were removed manually to maintain water quality and minimize interactions between feed and PS-MPs. Water and PS-MP concentrations were replenished in the treatment groups, whereas only water was renewed in the control group. Sampling was performed on days 10 and 20.

### 2.6 Preparation of sample

According to the American Veterinary Medical Association (AVMA) guidelines, adult *D. rerio* from each experimental group were euthanized using the hypothermic shock (rapid chilling) method. Fish were transferred to ice-chilled water maintained at 2 °C–4 °C and monitored until the loss of normal orientation and opercular movements. Following the cessation of opercular movement, the fish were maintained under the hypothermic condition for a minimum of 10 min to ensure death (Leary et al., 2020). The euthanized fish were then immediately processed for blood and tissue collection. Following euthanasia, blood samples were collected using 40-unit insulin syringes fitted with 32-gauge needles, which had been pre-chilled and coated with a solution of 2% EDTA and heparin (Beparine R heparin sodium, IP 1000 IU/mL derived from bovine intestinal mucosa and containing 0.15% w/v chlorocresol as an IP preservative). The entirety of the collected blood was utilized for hematological assessments. Subsequently, the principal organs, including gills, gut, and brain, were excised and preserved separately in Eppendorf vials for the purpose of analyzing targeted biomarkers and histological structures. Tissue samples (100 mg) were homogenized using a Teflon homogenizer in Tris-HCl buffer (pH 7.5). The resulting mixture was centrifuged for 15 min at 8,500 rcf and 4 °C. Thereafter, the supernatant was transferred into Eppendorf tubes for biomarker evaluation.

### 2.7 Hematological studies (RBC and WBC)

RBC and WBC counts were determined using a Burker hemocytometer following the method of Rusia and Sood (1992). Blood samples were immediately diluted (1:100) with Hayem’s solution (Witeska et al., 2022), and cell counts were performed under a Delta Optical Evolution 300 microscope at 200× (RBCs) and 400× (WBCs). Cell counts were calculated using standard hematological formulas (Bomski, 1983).

$$Total\;Cells=\frac{Number\;of\;Cells\;Counted\;\left(N\right)\times Dilution\;Factor}{Area\;Counted\;\left(mm^{2}\right)\times Depth\;of\;Chamber\;\left(mm\right)\;}$$

### 2.8 Plasma glucose

Plasma glucose was estimated by the O-toluidine method (Cooper et al., 1970). Briefly, 5 mL of O-toluidine reagent was mixed with 0.1 mL of plasma sample or glucose standard. The reaction mixture was heated in a boiling water bath for 10 min, cooled to room temperature, and absorbance was measured at 630 nm using a UV–Visible spectrophotometer. Plasma glucose concentration was calculated using the glucose standard.

$$Plasma\;Glucose\;\left(mg/dL\right)=\frac{O.D.\;of\;the\;sample}{O.D.\;ofthe\;Standard\;}\times 100$$

### 2.9 Plasma protein

Plasma protein was estimated according to the method of Lowry et al. (1951). Solution A was prepared by dissolving 2.0 g sodium carbonate in 100 mL of 0.1 N NaOH, while Solution B consisted of 5.0 g CuSO_4_ dissolved in 100 mL of 1% sodium potassium tartrate. The working reagent (Solution C) was prepared by mixing 50 mL of Solution A with 1 mL of Solution B. The Folin–Ciocalteu reagent was prepared by diluting 1 mL of Folin–phenol reagent with 1 mL of double-distilled water. For analysis, 0.1 mL of plasma was mixed with 0.9 mL of distilled water and 5 mL of Solution C, incubated for 10 min, followed by the addition of 0.5 mL of diluted Folin–Ciocalteu reagent. After 15 min of incubation, the absorbance was measured at 720 nm using a UV–Visible spectrophotometer, and bovine serum albumin (BSA) was used as the protein standard.

$$Plasma\;Protein\;\left(mg/mL\right)=\frac{O.D.\;of\;the\;sample}{O.D.\;ofthe\;Standard\;}\times Standard\;concentration\times Total\;Dilution\;Factor$$

### 2.10 Analysis of biochemical biomarkers

#### 2.10.1 Superoxide dismutase (SOD)

SOD estimation was performed by following the protocol of Kostyuk and Potapovich (1989). Enzyme activity was determined in an assay mixture containing 2.8 mL of (50 mM Tris Base, 10 mM EDTA; pH 8.2), 100 µL of 15 mM pyrogallol, and 100 µL of supernatant. The optical density was measured at 470 nm at 1 min intermissions for 3 min using a UV–Vis spectrophotometer, and the Unit is IU mg/protein.

$$SOD\;\left(IU/mg\;Protein\right)=\frac{\%\;inhibition}{Pyragallol\left(50\right)\;}\times \frac{Assay\;Volume\;\left(2.9\;mL\right)}{Sample\;Volume\;\left(0.1\;mL\right)}\times \frac{Dilution\;factor\;\left(1\right)}{mg\;of\;Protein}$$

#### 2.10.2 Glutathione-S-transferase (GST)

GST analysis was estimated according to the method of Habig et al. (1974). The reaction mixture contained 2.690 µL of 0.1 M PBS buffer, 10 µL of 15 mM CDNB, 200 µL of 1 mM GSH, 100 µL of supernatant and it was kept in an incubator for 30 min at 27 °C ± 2 °C. The enzymatic reaction was determined at 340 nm using a UV–Visible spectrophotometer and the measuring unit of µmole CDNB-GSH conjugate formed/min/mg protein.

$$GST\;\left(U/mg\right)=\frac{O.D.\;of\;the\;sample\times Total\;Volume\;of\;assay\left(3\;mL\right)}{Reaction\;Time\;\left(30\;\min\right)\times Sample\;Volume\;\left(0.1\;mL\right)\times CDNB\;coefficient\in \left(9.6\right)\times mg\;of\;Protein}$$

#### 2.10.3 Glutathione peroxidase (GPx)

According to Mohandas et al. (1984), the activity of glutathione peroxidase was measured. The entire 3 mL reaction solution contained 100 μL of 1 mM EDTA, 2.49 mL of 0.1 M PBS (pH 7.4), 100 μL of 1 mM sodium azide, 50 μL of 1 IU/mL glutathione reductase, 100 μL of 1 mM GSH, 100 μL of 0.2 mM NADPH, 10 μL of 0.25 mM H_2_O_2_, 100 μL of supernatant. At room temperature, the loss of NADPH is detected at 340 nm. The molar extinction coefficient of 6.22 × 10^3^ M^-1^cm^3^ was used to estimate the enzyme level as µ moles GSH oxidized/min/mg protein.

$$GPx\;\left(µmol\;GSH\;oxidized/\min/mg\;protein\right)=\frac{O.D.\;of\;the\;sample/3\;\min}{\in \left(6.22\times 10^{3}\right)\times d\left(1\right)}\times \frac{Total\;Volume\;of\;assay\left(3mL\right)}{Total\;volume\;of\;sample\left(0.1mL\right)}\times Unit\;Conversion\;Factor\times Stoichiometry\;Factor$$

#### 2.10.4 Lipid peroxidation (LPO)

The estimation of LPO was followed by the method of Devasagayam and Tarachand (1987). Malondialdehyde (MDA), an end product of lipid peroxidation, reacts with thiobarbituric acid (TBA) to form a pink colored complex (TBA-malondialdehyde adducts). The aliquots containing 0.1 mL of supernatant, 0.5 mL Tris HCL buffer (pH 8.2), and 0.15 mL of 10 mM KH_2_ PO_4_ and 0.25 mL of distilled water were incubated for 20 min at 37 °C. The reaction was terminated by adding 1.0 mL of 10% TCA to the aliquots and shaking well. Then 0.75 mL of TBA was added to it. The test tubes were kept in a boiling water bath for 10 min and centrifuged at 5,000 rpm for 10 min. The pink color developed was measured at 532 nm, and the unit of n mol MDA formed/mg protein.

$$LPO\;\left(nmol\;MDA/mg\;protein\right)=\frac{O.D.\;of\;the\;sample\times Total\;Volume\;of\;assay\left(3\;mL\right)\times 1000}{Sample\;Volume\;\left(0.1\;mL\right)\times \in \left(1.56\times 10^{5}M^{-1}cm^{-1}\right)\times mg\;of\;Protein}$$

#### 2.10.5 Acetylcholinesterase (AChE)

The method of Ellman et al. (1961) was used to estimate AChE. The reaction mixture contained 100 μL of supernatant, 2.86 mL of 0.1 M phosphate buffer (pH 8.0), 10 μL of DTNB, and 30 μL of Acetylthiocholine Iodide solution, which was incubated at room temperature for 30 min. The enzymatic reaction was determined at 412 nm using a UV–Visible spectrophotometer. The quantity of AChE activity needed to produce 1.0 nmol of choline per minute is one milliunit.

$$AChE\;\left(nmol/\min/mg\;protein\right)=\frac{O.\;D.\;of\;the\;sample\times Total\;volume\;of\;assay\left(3\;mL\right)\times 1000}{\in \left(13.6\right)\times Incubation\;time\left(30\;\min\right)\times Volume\;of\;sample\;\left(0.1\;mL\right)\times mgof\;protein}$$

### 2.11 Histopathological studies

The gills, gut, and brain of the fish specimens underwent processing for histopathological examination (Borges et al., 2020). The tissues were cleansed with a 0.9% physiological saline solution and subsequently preserved in a 10% formaldehyde solution. Following this, paraffin wax was applied, and the tissues were sectioned into transverse slices (5 μm) utilizing a microtome (Leica-cryocut1800, Germany). Eosin and hematoxylin were employed to stain the mounted tissues. The tissues were scrutinized under a light microscope at a magnification of ×10 (Lawrence and Mayo: NLCD-307).

### 2.12 Statistical analysis

Homogeneity of variance was assessed using Levene’s test before ANOVA. A formal test of normality was not performed; therefore, the assumption of normality could not be independently verified and is acknowledged as a limitation of the statistical analysis. Data were expressed as mean ± SD. Univariate analysis incorporating Tukey’s *post hoc* test was employed to evaluate the effects of treatment groups, exposure days, and their interactions. The three replicate groups were maintained independently, and statistical comparisons were performed using group-level observations obtained from the experimental replicates. Effect sizes were reported as eta squared (*η* ^*2*^) along with observed power. A *p*-value < 0.05 was considered statistically significant. Statistical analysis was performed using SPSS version 22.0 (SPSS Inc.). Table 2 presents the statistical results for hematological, biochemical, antioxidant, and neurotoxicity parameters in *D. rerio* exposed to PS-MPs. All raw data (Dataset S1) are provided in the Supplementary Material.

**Table 2:** Statistical results in D. rerio exposed to PS-MPs.

Parameter	Source	*F Value*	*P* < 0.05	Partial *η^2^*	Observed power	Adjusted *R^2^* Value
RBC	Days Groups Interactions	*F*_(1,48)_ = 12.130 *F*_(2,48)_ = 48.516 *F*_(2,48)_ = 0.207	0.001 0.000 0.814	0.202 0.669 0.009	0.927 1.000 0.080	0.664
WBC	Days Groups Interactions	*F*_(1,48)_ = 0.010 *F*_(2,48)_ = 322.802 *F*_(2,48)_ = 21.216	0.920 0.000 0.000	0.000 0.931 0.469	0.051 1.000 1.000	0.928
Plasma glucose	Days Groups Interactions	*F*_(1,48)_ = 719.780 *F*_(2,48)_ = 2746.307 *F*_(2,48)_ = 123.094	0.000 0.000 0.000	0.937 0.991 0.837	1.000 1.000 1.000	0.992
Plasma protein	Days Groups Interactions	*F*_(1,48)_ = 104.632 *F*_(2,48)_ = 1201.077 *F*_(2,48)_ = 103.482	0.000 0.000 0.000	0.686 0.980 0.812	1.000 1.000 1.000	0.981
LPO gill	Days Groups Interactions	*F*_(1,48)_ = 576.621 *F*_(2,48)_ = 2023.451 *F*_(2,48)_ = 107.266	0.000 0.000 0.000	0.923 0.998 0.817	1.000 1.000 1.000	0.989
SOD gill	Days Groups Interactions	*F*_(1,48)_ = 1228.423 *F*_(2,48)_ = 27958.972 *F*_(2,48)_ = 751.885	0.000 0.000 0.000	0.962 0.999 0.969	1.000 1.000 1.000	0.999
GST gill	Days Groups Interactions	*F*_(1,48)_ = 12753.120 *F*_(2,48)_ = 104924.413 *F*_(2,48)_ = 4409.320	0.000 0.000 0.000	0.996 1.000 0.995	1.000 1.000 1.000	1.000
GPx gill	Days Groups Interactions	*F*_(1,48)_ = 26901.048 *F*_(2,48)_ = 96726.735 *F*_(2,48)_ = 16930.573	0.000 0.000 0.000	0.998 1.000 0.999	1.000 1.000 1.000	1.000
SOD gut	Days Groups Interactions	*F*_(1,48)_ = 9068.526 *F*_(2,48)_ = 16504.173 *F*_(2,48)_ = 2908.688	0.000 0.000 0.000	0.995 0.999 0.992	1.000 1.000 1.000	0.999
GST gut	Days Groups Interactions	*F*_(1,48)_ = 2947.220 *F*_(2,48)_ = 7555.323 *F*_(2,48)_ = 848.930	0.000 0.000 0.000	0.984 0.997 0.973	1.000 1.000 1.000	0.997
GPx gut	Days Groups Interactions	*F*_(1,48)_ = 417911.522 *F*_(2,48)_ = 363359.001 *F*_(2,48)_ = 169138.372	0.000 0.000 0.000	1.000 1.000 1.000	1.000 1.000 1.000	1.000
AChE brain	Days Groups Interactions	*F*_(1,48)_ = 740.877 *F*_(2,48)_ = 7550.498 *F*_(2,48)_ = 226.819	0.000 0.000 0.000	0.939 0.997 0.904	1.000 1.000 1.000	0.997
SOD brain	Days Groups Interactions	*F*_(1,48)_ = 218435.331 *F*_(2,48)_ = 147318.730 *F*_(2,48)_ = 64977.887	0.000 0.000 0.000	1.000 1.000 1.000	1.000 1.000 1.000	1.000
GST brain	Days Groups Interactions	*F*_(1,48)_ = 43.321 *F*_(2,48)_ = 408.541 *F*_(2,48)_ = 101.683	0.000 0.000 0.000	0.474 0.945 0.809	1.000 1.000 1.000	0.952
GPx brain	Days Groups Interactions	*F*_(1,48)_ = 807253.213 *F*_(2,48)_ = 430067.089 *F*_(2,48)_ = 212807.281	0.000 0.000 0.000	1.000 1.000 1.000	1.000 1.000 1.000	1.000

## 3 Results

### 3.1 Characterization of PS-MPs

#### 3.1.1 SEM analysis

The morphology and surface characteristics of the PS-MPs were examined by SEM (Figures 1A,B). At 300× magnification, the particles exhibited predominantly irregular and fragmented structures with frayed and jagged edges (Figure 1A). Higher magnification (2000×) revealed rough and folded surface features with heterogeneous structural organization (Figure 1B). The SEM observations therefore indicated irregular, non-uniform PS-MP morphology with micrometre-scale dimensions. The observed particle dimensions ranged approximately from 1 to 200 µm across the examined micrographs.

**FIGURE 1:**
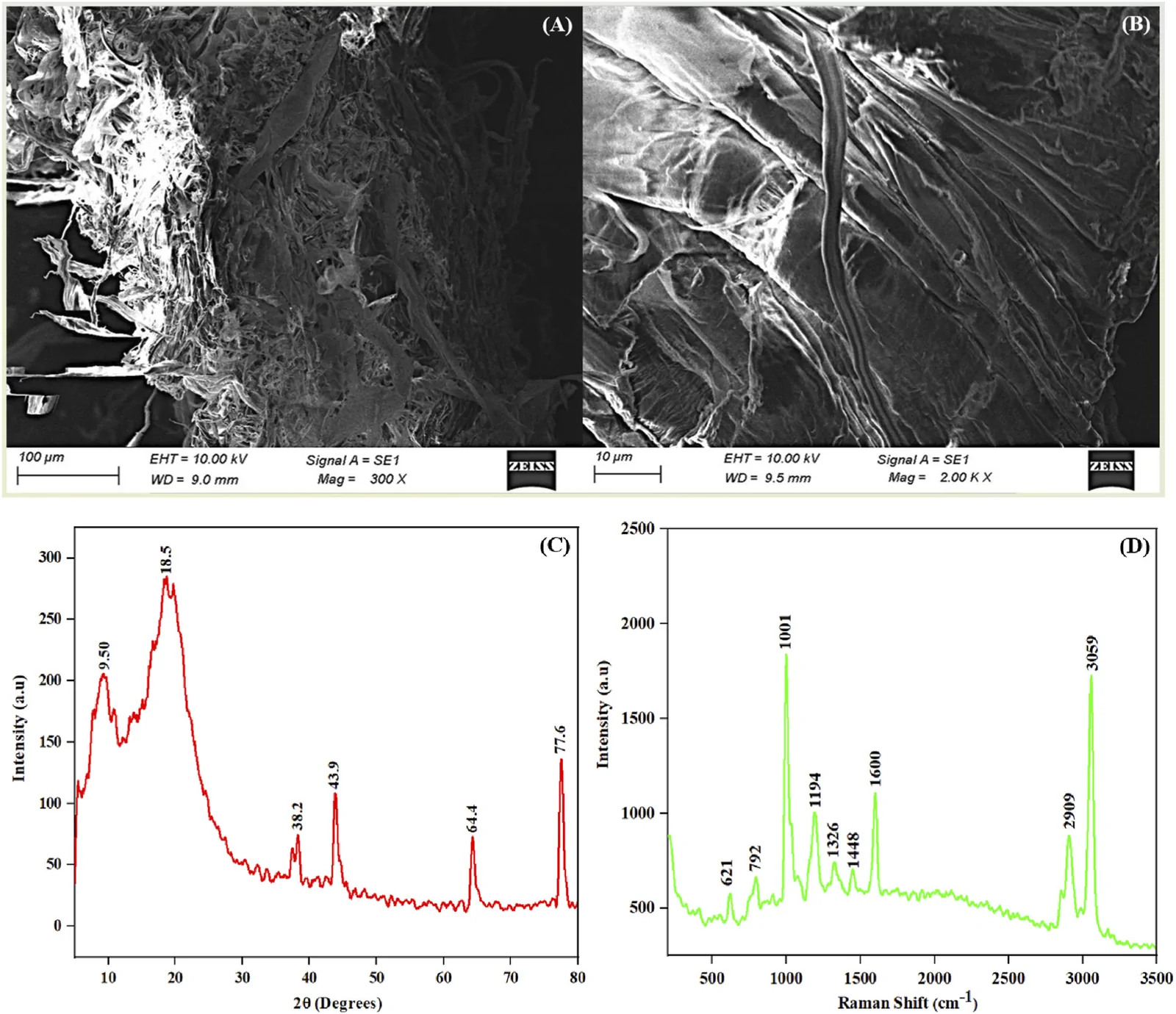
Characterization of PS-MPs. **(A,B)** SEM image showing the irregular morphology of PS-MPs. **(C)** X-ray diffraction of PS-MPs. **(D)** Raman Spectroscopy of PS-MPs.

#### 3.1.2 XRD diffraction

The XRD analysis was mainly performed to determine the crystallinity of PS-MPs. XRD patterns of pure PS-MPs were obtained over the range 10^∘^ −80^∘^ (Figure 1C). Several peaks were obtained at 2θ lattice values such as 9.50^0^, 18.5^0^, 38.2^0^, 43.9^0^, 64.4^0,^ and 77.6^0^. The diffraction peaks were observed in 2θ angles of 9.50^0^ and 18.5^0^ are two prominent peaks. The 2θ = 9.50^0^ indicates the presence of amorphous material in the PS polymer, while the peaks at 18.5^0^ indicate the presence of crystalline polymer material.

#### 3.1.3 Raman spectroscopy analysis

In Figure 1D, several peaks were obtained in the range between 500-3,500 cm^-1^ to confirm the PS-MPs, such as 621 cm^-1^, 792 cm^-1^, 1,001 cm^-1^, 1,194 cm^-1^, 1,326 cm^-1^, 1,448 cm^-1^, 1,600 cm^-1^, 2,909 cm^-1^, 3,059 cm^-1^. The prominent band at 1,001 cm^-1^ is characteristic of aromatic ring vibrations in polystyrene, while the band at 1,600 cm^-1^ corresponds to aromatic C=C stretching. The bands at 2,909 and 3,059 cm^-1^ are associated with aliphatic C–H and aromatic C–H stretching vibrations, respectively. Additional bands at 621, 792, 1,194, 1,326, and 1,448 cm^-1^ are attributable to aromatic ring deformation and C–H vibrational modes, supporting the identification of the material as PS.

#### 3.1.4 DLS analysis

The DLS analysis of PS-MPs revealed an average hydrodynamic particle size (Z-average) of 1,115.5 nm in water at 25.0 °C, with a polydispersity index (PDI) of 0.569, indicating a highly heterogeneous and broad particle size distribution (Figure 2A). The autocorrelation function (ACF) curve in Figure 2B exhibited a gradual and extended decay profile across a broad logarithmic time scale. This non-exponential decay is consistent with a highly polydisperse suspension containing particles and aggregates with different diffusion rates. The discrepancy between the micrometre-scale dimensions observed by SEM and the DLS-derived hydrodynamic diameter reflects the different measurement principles of the two techniques and the influence of particle aggregation in aqueous suspension.

**FIGURE 2:**
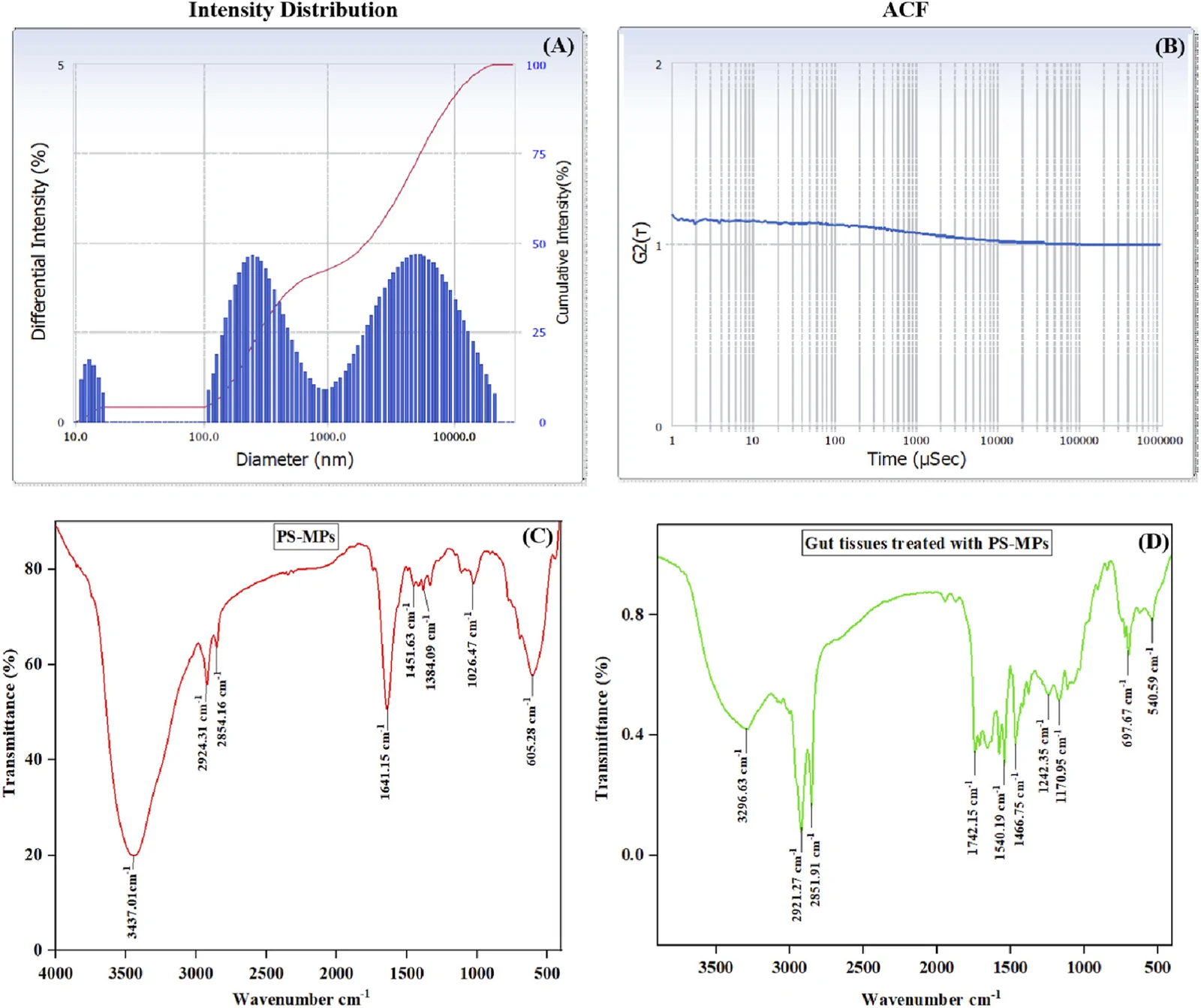
Characterization of PS-MPs. **(A,B)** DLS determined the aggregation of PS-MPs in water with autocorrelation function (ACF). **(C)** FTIR spectra of PS-MPs. **(D)** FTIR analysis of *Danio rerio* gut tissues treated with PS-MPs.

#### 3.1.5 FTIR analysis of PS-MPs and *Danio rerio* gastrointestinal tract exposure to PS-MPs

FTIR spectra of PS-MPs samples are shown in Figure 2C. The recorded major peaks at 3,437.01 cm^-1^, 2,924.16 cm^-1^, 2,854.16 cm^-1^, 1,641.15 cm^-1^, 1,453.63 cm^-1^, 1,384.09 cm^-1^, 1,026.47 cm^-1^ and 605.28 cm^-1^. The prominent aliphatic C-H stretching peaks at 2,924.16 cm^-1^ and 2,854.16 cm^-1^, alongside a CH_2_ bending peak at 1,453.63 cm^-1,^ correspond to the backbone of the PS-MPs. The O-H stretching band at 3,437.01 cm^-1^ and the peak at 1,641.15 cm^-1^ indicate surface oxidation and hydroxyl accumulation of PS-MPs. Additional peaks such as 1,384.09 cm^-1^, 1,026.47 cm^-1^ indicate structural confirmation and the aromatic peak at 605.28 cm^-1^.

FTIR spectra of PS-MPs in the gut tissue of fish are shown in Figure 2D. Several peaks were observed such as 3,296.63 cm^-1^, 2,921.27 cm^-1^, 2,851.91 cm^-1^, 1742.15 cm^-1^, 1,540.19 cm^-1^, 1,466.75 cm^-1^, 1,242.35 cm^-1^, 1,170.95 cm^-1^, 697.67 cm^-1^ and 540.59 cm^-1^. The prominent peak at 3,296.63 cm^-1^ indicates the O-H stretching band; the peaks at 1,540.19 cm^-1^ and 1,242.35 cm^-1^ confirm the Amide II protein and Amide III groups; 1,466.75 cm^-1^ denotes CH_2_ bending vibration. The strong peaks at 2,921.27 cm^-1^ and 2,851.91 cm^-1^ represent C-H stretching vibrations, indicating the backbone of the accumulated PS-MPs. The sharp peaks at 1742.15 cm^-1^ and 1,170.95 cm^-1^ represent C=O carbonyl stretching and C-O stretching vibrations. The distinct important peak at 697.67 cm^-1^ corresponds to the aromatic C-H bending band and these peaks provide supporting evidence for the occurrence of PS-related material in the gastrointestinal tract following exposure.

### 3.2 Acute toxicity test-LC_50_ values and morphogenetic variations of *Danio rerio*

No mortality occurred in the control group during the acute toxicity test (96 h). PS-MPs exposure induced mortality in the zebrafish. Acute exposure to PS-MPs resulted in a concentration-dependent increase in mortality in *D. rerio*. Mortality increased from 30% at 50 μg/L to 50%, 63.33%, 83.33%, and 100% at 100, 150, 200, and 250 μg/L, respectively, whereas no mortality was observed in the control group. Based on probit analysis of the concentration–mortality relationship, the 96-h LC_50_ of PS-MPs was estimated to be 89.1 μg/L (95% CI: 69.234–107.051 μg/L). After 96 h, several morphogenetic deformities were observed (Figures 3A,B) in the control and treated groups. In the control, Figure 3A shows healthy Mouth (M), Eye (E), Head (H), Operculum (O), Pectoral fin (PF), Lateral line (LL), Dorsal fin (DF), Anal fin (AF), Abdominal/Urogenital pore (AUP), and Caudal fin (CF) are observed. But the treated fish (Figure 3B) show disruption of the lateral line (DLL), Spinal deformity (SD), Severe abdominal distension (SAD), Disruption of the dorsal and anal fins (DDF & DAF), disruption and discoloration of the caudal fin (DCF).

**FIGURE 3:**
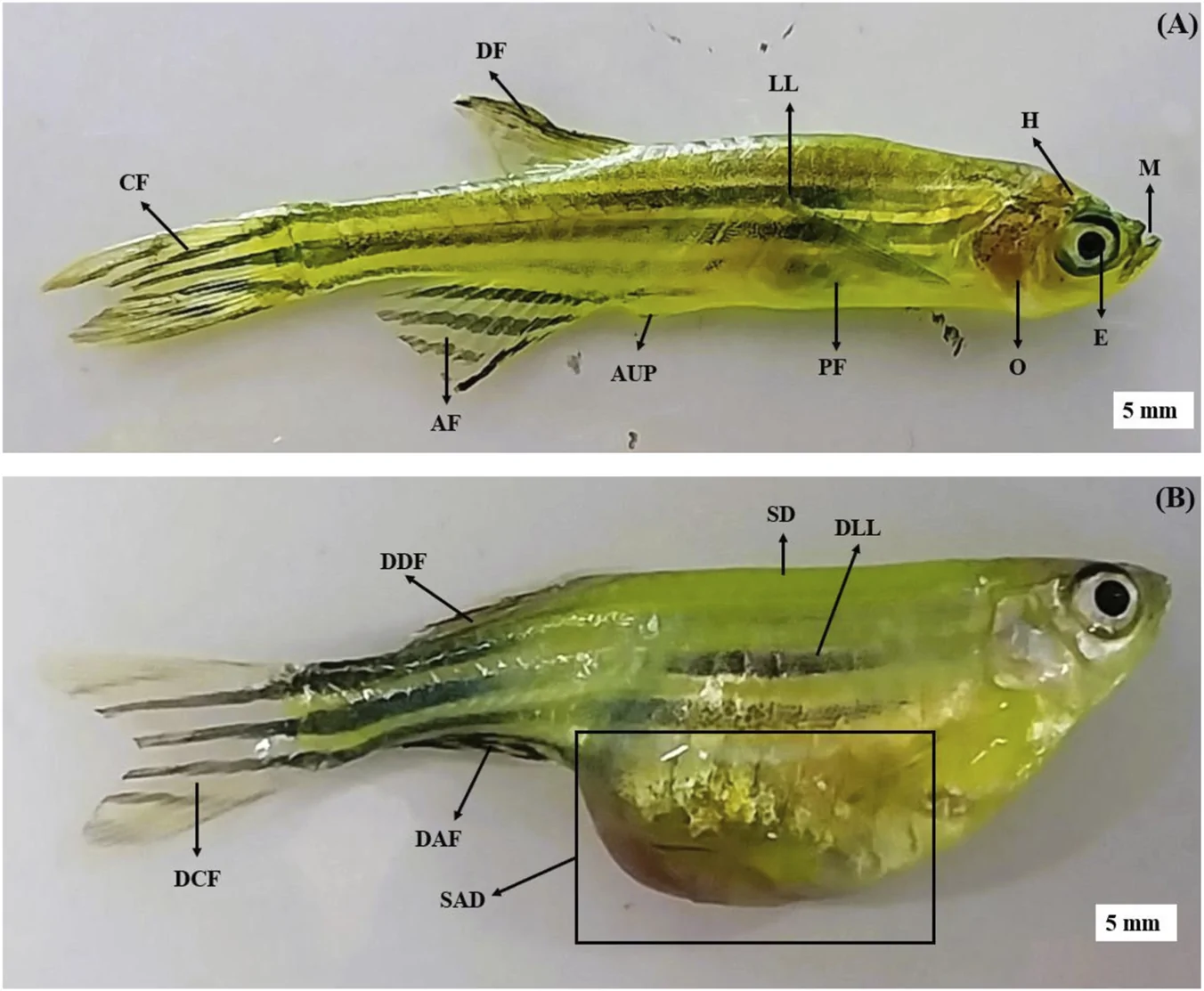
Morphogenetic variation of *Danio rerio* treated with PS-MPs. **(A)** Control–Mouth (M), Eye (E), Head (H), Operculum (O), Pectoral fin (PF), Lateral line (LL), Dorsal fin (DF), Anal fin (AF), Abdominal/Urogenital pore (AUP), Caudal fin (CF). **(B)** Treated–Disruption of Lateral line (DLL), Spinal Deformity (SD), Severe abdominal distension (SAD), Disruption of Dorsal and Anal fin (DDF and DAF), Disruption and discoloration of caudal fin (DCF).

### 3.3 Hematological studies

Figures 4A,B shows the hematological responses of *D. rerio* following PS-MP exposure. Compared with the control, RBC counts increased significantly (*P* < 0.05) from 2.79 ± 0.19 to 3.00 ± 0.27 × 10^6^ cells mm^-3^ in Treatment I and from 3.19 ± 0.24 to 3.51 ± 0.20 × 10^6^ cells mm^-3^ in Treatment II. In contrast, WBC counts decreased significantly from 6.62 ± 0.96 to 5.72 ± 0.96 × 10^3^ cells mm^-3^ in Treatment I and from 4.69 ± 0.63 to 3.21 ± 1.04 × 10^3^ cells mm^-3^ in Treatment II. The greatest changes were observed in Treatment II.

**FIGURE 4:**
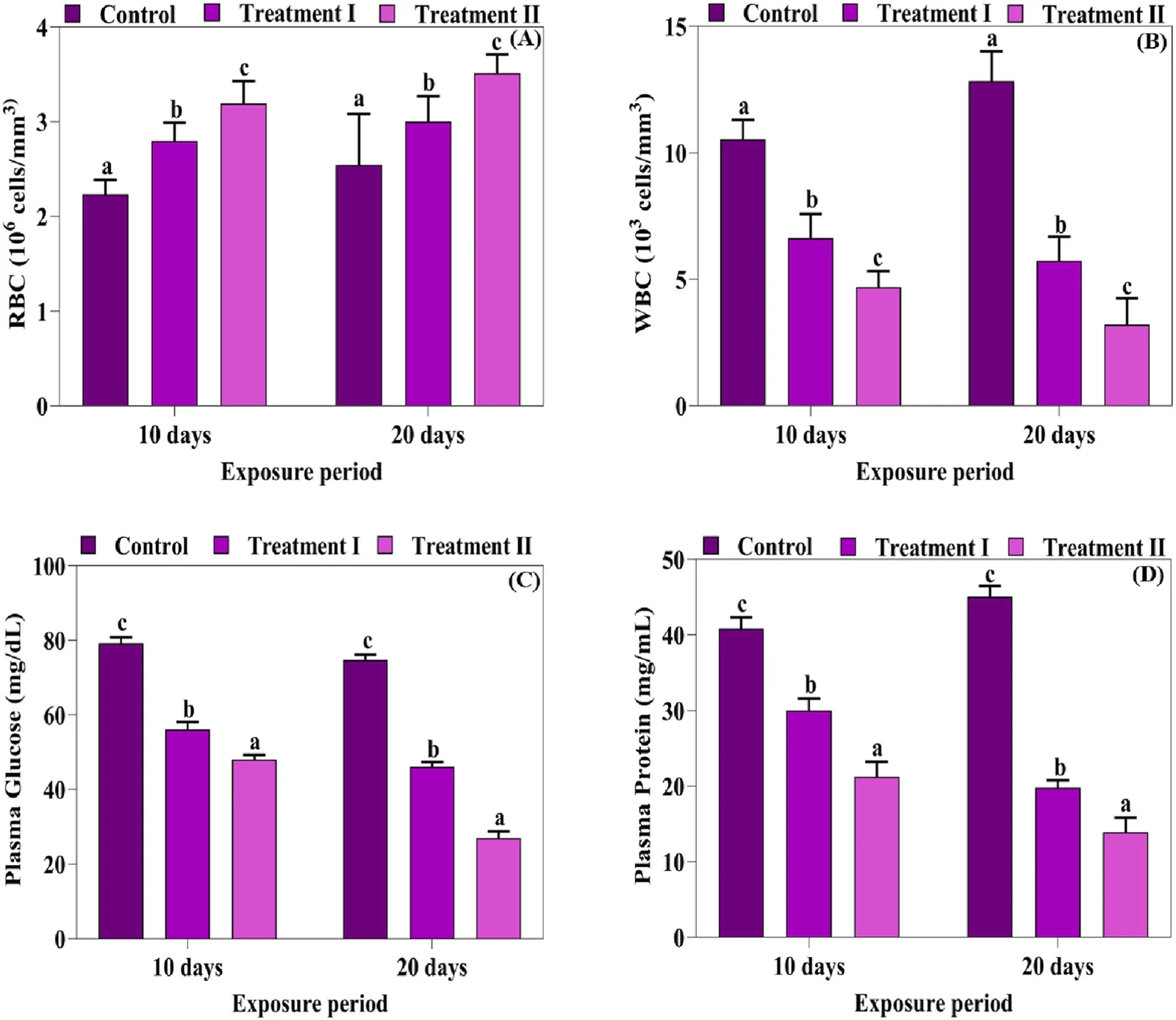
Hematological parameters and biochemical responses of *Danio rerio*. **(A)** RBC, **(B)** WBC, **(C)** Plasma glucose, **(D)** Plasma protein. Bars indicate Mean ± S.D (n = 9 fish/group). Distinct lowercase letters indicate significant differences between experimental groups as determined by (Univariate analysis) two-way ANOVA (with Tukey’s, Post-hoc) at *p* < 0.05. Treatment I-2.5 μg/L and Treatment II-25 μg/L.

### 3.4 Plasma glucose and plasma protein

Figures 4C,D shows the plasma biochemical responses of *D. rerio* following PS-MP exposure. Plasma glucose levels decreased significantly (*P* < 0.05) from 56.09 ± 1.98 to 46.05 ± 1.28 mg/dL in Treatment I and from 48.03 ± 1.27 to 26.96 ± 1.84 mg/dL in Treatment II compared with the control (79.10 ± 1.74 to 74.70 ± 1.48 mg/dL). Similarly, plasma protein levels declined significantly from 30.02 ± 1.57 to 19.80 ± 0.91 mg/mL in Treatment I and from 21.26 ± 1.97 to 13.90 ± 1.91 mg/mL in Treatment II relative to the control (40.80 ± 1.52 to 45.05 ± 1.44 mg/mL), with the greatest reductions observed in Treatment II.

### 3.5 Antioxidant and oxidative stress analysis

#### 3.5.1 Enzymatic changes observed in *Danio rerio* gill tissues

Figures 5A–D presents the antioxidant responses in the gill tissues of *D. rerio* following PS-MP exposure. Compared with the control, LPO, SOD, and GPx activities increased significantly (*P* < 0.05), whereas GST activity decreased significantly in both treatment groups. LPO levels increased from 16.41 ± 1.76 to 27.34 ± 0.70 nmol MDA formed mg^-1^ protein in Treatment I and from 24.77 ± 0.80 to 33.04 ± 1.28 nmol MDA formed mg^-1^ protein in Treatment II. SOD activity ranged from 87.29 ± 1.51 to 78.40 ± 0.87 IU mg^-1^ protein in Treatment I and 132.27 ± 1.93 to 101.70 ± 1.06 IU mg^-1^ protein in Treatment II. GST activity decreased from 0.104 ± 0.001 to 0.074 ± 0.001 μmol CDNB–GSH conjugate min^-1^ mg^-1^ protein in Treatment I and from 0.098 ± 0.001 to 0.022 ± 0.002 μmol CDNB–GSH conjugate min^-1^ mg^-1^ protein in Treatment II. Conversely, GPx activity increased from 146.74 ± 1.24 to 170.15 ± 1.19 μmol GSH oxidized min^-1^ mg^-1^ protein in Treatment I and from 138.83 ± 1.97 to 290.49 ± 1.62 μmol GSH oxidized min^-1^ mg^-1^ protein in Treatment II. The most pronounced changes in all antioxidant biomarkers were observed in Treatment II.

**FIGURE 5:**
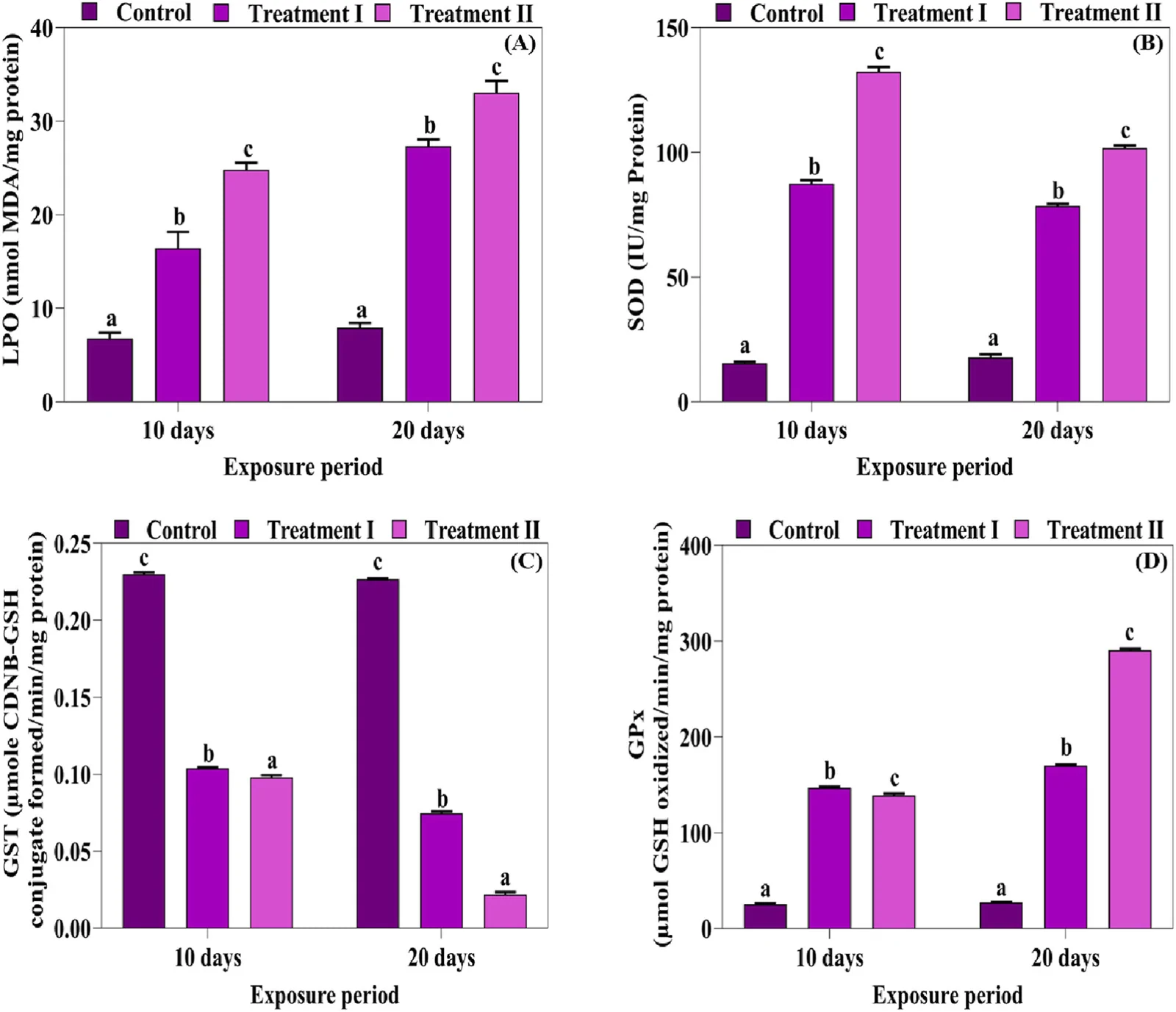
Antioxidant activity in the gill of *Danio rerio*. **(A)** Lipid peroxidation (LPO), **(B)** Superoxide dismutase (SOD), **(C)** Glutathione -S- transferase (GST), **(D)** Glutathione peroxidase (GPx). Bars indicate Mean ± S. D (n = 9 fish/group). Distinct lowercase letters indicate significant differences between experimental groups as determined by (Univariate analysis) two-way ANOVA (with Tukey’s, Post-hoc) at *p* < 0.05. Treatment I-2.5 μg/L and Treatment II-25 μg/L.

#### 3.5.2 Enzymatic changes observed in *Danio rerio* gut tissues

PS-MP exposure markedly altered the antioxidant status of the gut tissues in *D. rerio* (Figures 6A–C). SOD activity was significantly elevated (*P* < 0.05), increasing from 38.48 ± 1.66 to 77.47 ± 1.79 IU mg^-1^ protein in Treatment I and from 56.28 ± 1.52 to 133.50 ± 1.84 IU mg^-1^ protein in Treatment II. In contrast, GST activity declined progressively, reaching 0.056 ± 0.003 and 0.029 ± 0.003 μmol CDNB–GSH conjugate min^-1^ mg^-1^ protein in Treatments I and II, respectively. GPx activity showed a pronounced increase, ranging from 67.15 ± 1.05 to 210.57 ± 1.02 μmol GSH oxidized min^-1^ mg^-1^ protein in Treatment I and from 118.02 ± 0.80 to 501.65 ± 1.49 μmol GSH oxidized min^-1^ mg^-1^ protein in Treatment II. Overall, the antioxidant responses were more pronounced in fish exposed to 25 μg/L PS-MPs than in those exposed to 2.5 μg/L.

**FIGURE 6:**
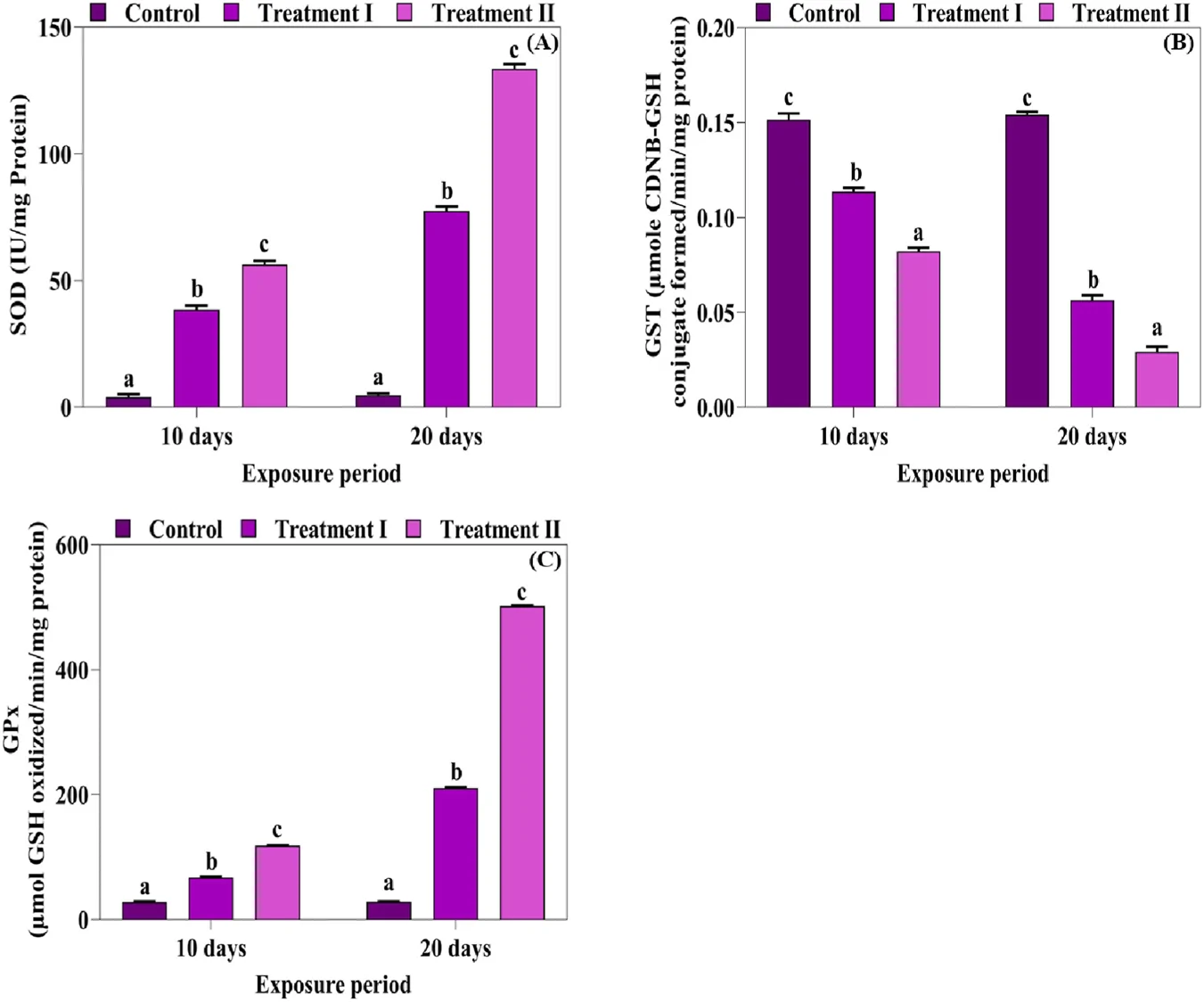
Antioxidant activity in the gut of *Danio rerio*. **(A)** Superoxide dismutase (SOD), **(B)** Glutathione -S- transferase (GST), **(C)** Glutathione peroxidase (GPx). Bars indicate Mean ± S. D (n = 9 fish/group). Distinct lowercase letters indicate significant differences between experimental groups as determined by (Univariate analysis) two-way ANOVA (with Tukey’s, Post-hoc) at *p* < 0.05. Treatment I-2.5 μg/L and Treatment II-25 μg/L.

#### 3.5.3 Enzymatic and AChE activity observed in *Danio rerio* brain tissues

Neurochemical and antioxidant biomarkers in the brain were significantly affected by PS-MP exposure (Figures 7A–D). A marked inhibition of AChE activity (*P* < 0.05) was evident in both treatment groups, decreasing to 27.23 ± 1.40 and 8.33 ± 1.43 μmol min^-1^ mL^-1^ in Treatments I and II, respectively, after 20 days. Simultaneously, SOD activity increased markedly, reaching 280.93 ± 1.94 IU mg^-1^ protein in Treatment I and 484.65 ± 1.65 IU mg^-1^ protein in Treatment II. In contrast, GST activity remained below the control throughout the exposure period, with the lowest level (0.081 ± 0.007 μmol CDNB–GSH conjugate formed min^-1^ mg^-1^ protein) observed in Treatment I. GPx activity was also markedly enhanced, attaining 775.03 ± 1.22 and 771.00 ± 2.89 μmol GSH oxidized min^-1^ mg^-1^ protein in Treatments I and II, respectively, indicating a pronounced antioxidant response to PS-MP-induced oxidative stress.

**FIGURE 7:**
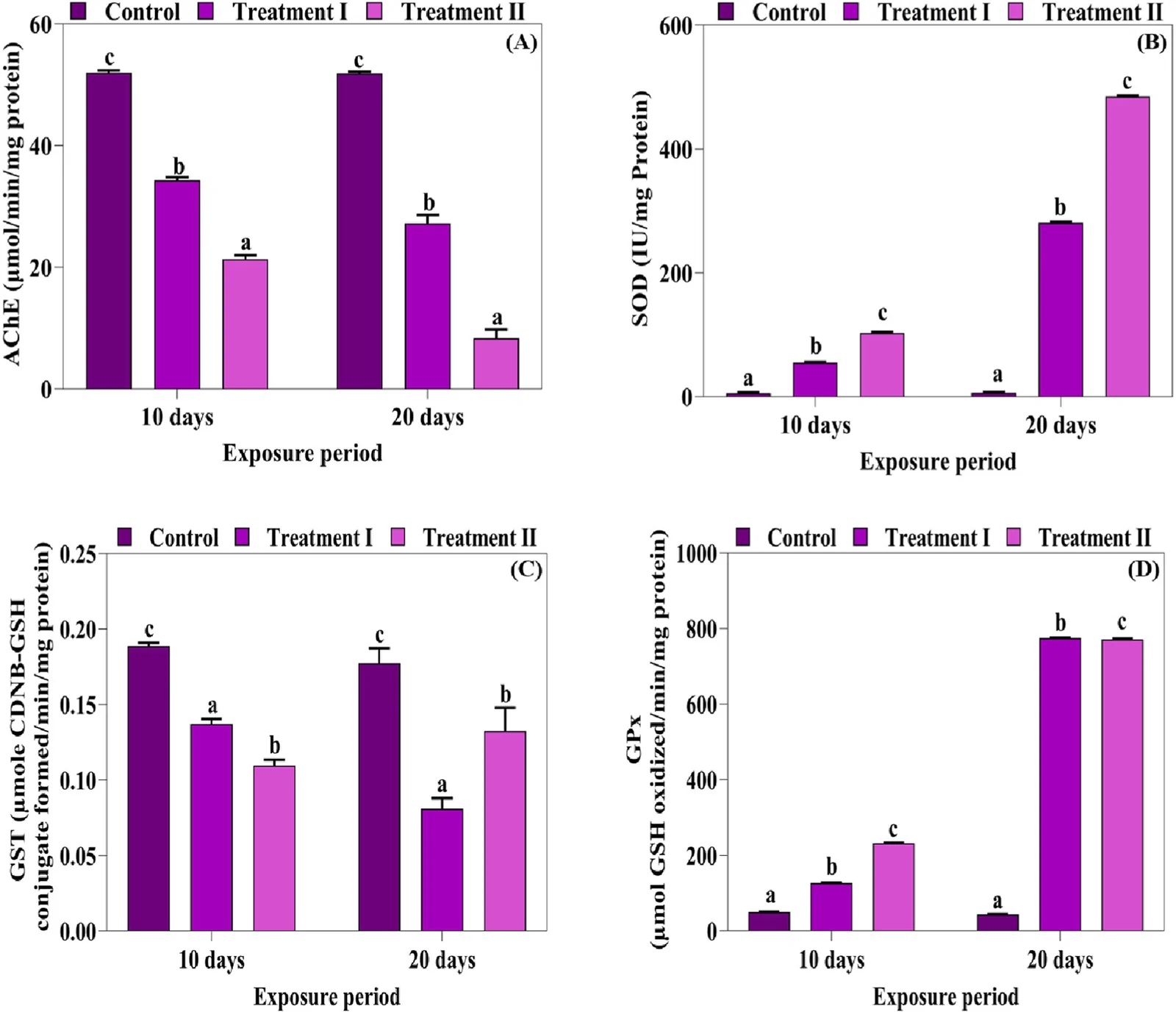
AChE and antioxidant activities in the brain of *Danio rerio*. **(A)** Acetylcholinesterase activity AChE), **(B)** Superoxide dismutase (SOD), **(C)** Glutathione -S- transferase (GST), **(D)** Glutathione peroxidase (GPx). Bars indicate Mean ± S. D (n = 9 fish/group). Distinct lowercase letters indicate significant differences between experimental groups as determined by (Univariate analysis) two-way ANOVA (with Tukey’s, Post-hoc) at *p* < 0.05. Treatment I-2.5 μg/L and Treatment II-25 μg/L.

### 3.6 Histological observations

The histological examination of *D. rerio* gill revealed severe damage in the control, Treatment I and Treatment II (Figures 8A–C). The control group exhibits normal gill rakers (GR), intact skeletal muscle (SM), Adipose Tissue (AT), Efferent Branchial arterioles (EBA), Afferent Branchial arterioles (ABA), uniform primary and secondary lamellae (SL), Chondrocyte Cells (CD). In contrast, Treatment I and II show prominent damage such as thinning of gill rakers (TGR), Disruption of submucosa, efferent and afferent branchial arterioles (DSM, DEBA, DABA), damage to chondrocyte cells, primary and secondary lamellae (DCD, DPL, DSL). Figures 8D–F shows histological damage of *D. rerio* gut tissues from control to treatment groups. The control group clearly shows well-organized intestinal villi (V), Muscle layer (ML), uniform mucosal epithelium (ME), serosa (S), lamina propria (LP), goblet cells (GC), lumen (L), and enterocytes (EC). In treated groups shows vacuolization of the muscular layer (VML), Epithelial lifting and sloughing (ELS), disorganization of the lamina propria (DLP), shortening and disruption of villi (SV&DV). Finally, histological damage was observed in the brain tissues of *D. rerio* (Figures 8G–I). The control image shows a normal molecular layer (ML) a closely arranged granular layer (GL) containing intact neurons, and normal blood vessels (BV). In Contrast, Treatment I clearly illustrates early degenerative changes, including edema (E), many haloid cells showing apoptotic pyknotic nuclei (PN), and initial granular layer dispersal (GD). Treatment II demonstrates highly disrupted tissues, leading to severe vacuolization (SV), cellular necrosis (CN) with fragmented nuclei, and granular layer disintegration (GLD).

**FIGURE 8:**
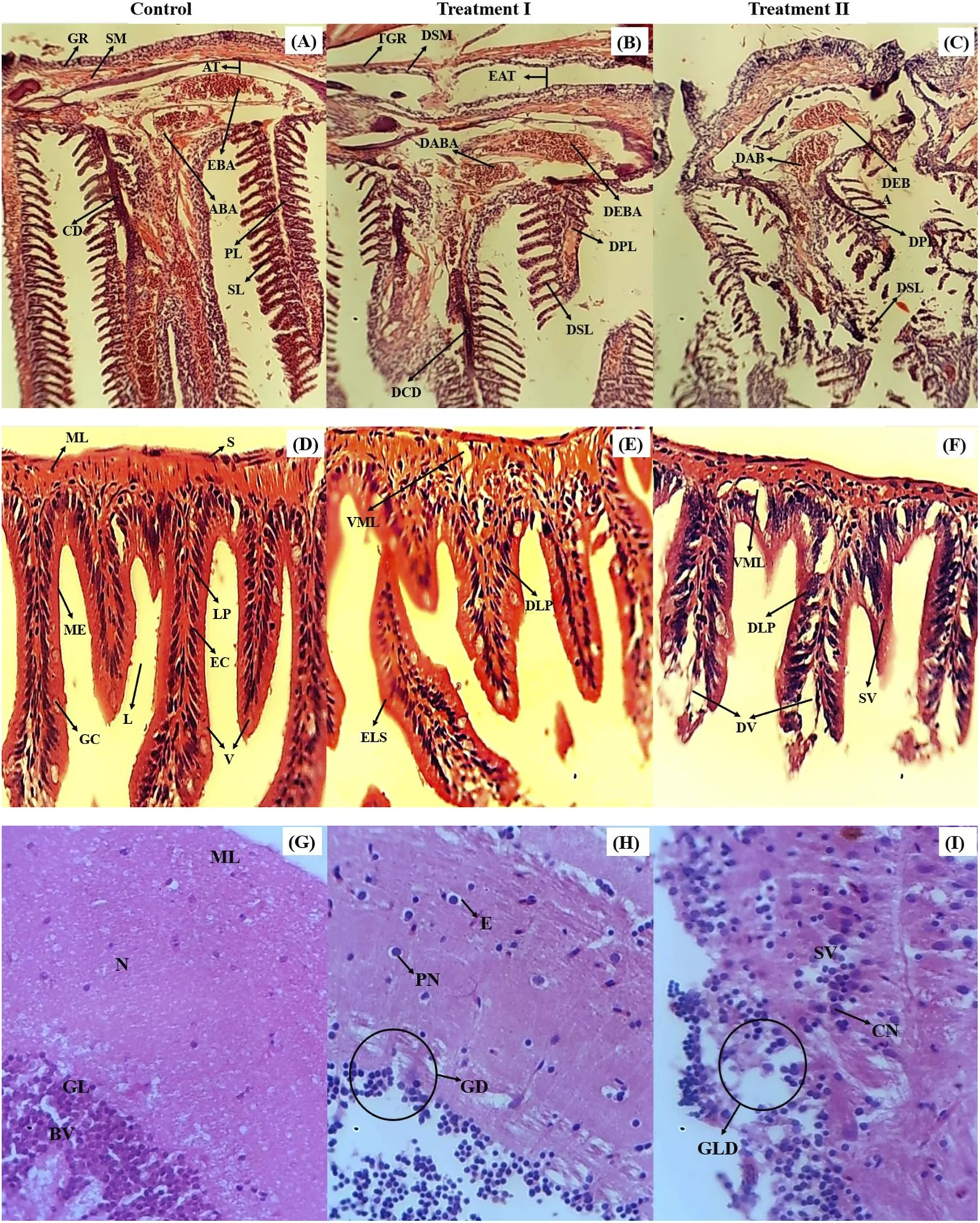
Histological observation of *Danio rerio* for 20 days. **(A–C)** Gill histology. **(A)** Control–GR (Gill Rakers), SM (Submucosa), AT (Adipose Tissue), EBA (Efferent Branchial Arterioles), ABA (Afferent Branchial Arterioles), PL (Primary Lamellae), SL (Secondary Lamellae), CD (Chondrocyte Cells). **(B,C)** Treated–TGR (Thinning of Gill Rakers), DSM, DEBA, DABA (Disruption of submucosa, efferent and afferent branchial arterioles), DCD, DPL, DSL (Damages chondrocyte cells, primary and secondary lamellae). **(D–F)** Gut histology. **(D)** Control–ML (Muscle layer), ME (Mucosal epithelium), S (Serosa), EC (Enterocytes), V (Villi), GC (Goblet Cells), LP (Lamina Propria), L (Lumen). **(E,F)** Treated–VML (Vacuolization of muscular layer), ELS (Epithelial lifting and sloughing), DLP (Disorganization of lamina propria), SV&DV (Shortening and disruption of villi). **(G–I)** Brain histology. **(G)** Control–ML (Molecular layer), N (Neurons), GL (Granular layer), BV (Blood vessels). **(H,I)** E (Edema), PN (Pyknotic nuclei), GD (Dispersal of granular layer), SV (Severe vacuolization), CN (Cellular necrosis).

## 4 Discussion

The current study sheds light on the dose- and time-dependent Sub-chronic toxicity of PS-MPs in *D. rerio*, which causes alterations in hematological, plasma glucose and protein, antioxidant, and neurotoxic indicators, as well as histopathological damage. These findings shed light on the toxicity patterns of PS-MPs that are particular to polymers and organoids, as well as their possible ecological concern in freshwater systems. Several characterizations were conducted, such as SEM, XRD, Raman spectroscopy, DLS, and FTIR. SEM observations revealed irregular and aggregated microplastic structures with micrometre-scale dimensions, with observed features ranging approximately from 1 to 200 µm. The XRD peaks at 9.50^0^ indicate amorphous material in the PS polymer, while the peaks at 18.5^0^ indicate the presence of crystalline polymer material. Previous reports have investigated the size, shape, and crystallinity of PS-MPs and their impact on artificial digestion (Wiedner and Polifka, 2020). The biological effects and ecological risks associated with microplastic exposure are influenced by particle characteristics, environmental persistence, exposure conditions, and interactions with aquatic organisms (Ansari et al., 2025).

Raman spectroscopic analysis was performed to verify the chemical composition and structural integrity of the MPs particles. Gao et al. (2023) already reported similar peaks such as 620, 795, 1,001, 1,029, 1,182, 1,448 and 1,603 cm^-1^ for PS-MPs in aqueous environments. The FTIR peaks, such as the aromatic C-H and aliphatic CH_2_ stretching vibrations, yielded intense bands just above and below 3,000 cm^-1^. The aromatic ring vibrations showed peaks at 1,602, 1,542, 1,494 and 1,444 cm^-1^. The peaks corresponding to CH2 bending modes were observed at 1,444 and 1,373 cm^-1^. The group of peaks below 1,000 cm^-1^ due to aromatic CH bending modes confirms the functional group of the PS-MPs, which is suitable for toxic studies (Vinodh et al., 2015).

The 96-h LC_50_ value of PS-MPs for *D. rerio* (89.1 μg/L) indicates acute toxicity at a relatively low concentration, with mortality increasing in a concentration-dependent manner. Based on the experimentally determined LC_50_, we selected sub-lethal concentrations of 2.5 and 25 μg/L for the 20-day exposure experiment to assess concentration-dependent physiological and biochemical responses. Although pristine microplastics are generally considered to exhibit relatively low acute lethality, the observed response may depend on particle characteristics, aggregation state, exposure conditions and organism-specific susceptibility. The present study did not specifically quantify additives, leachable compounds or residual styrene monomers; therefore, their contribution to the observed mortality cannot be confirmed. Physical interactions of PS-MPs with the gill and gastrointestinal surfaces, together with associated physiological stress and oxidative imbalance, may contribute to the observed toxicity. Similar LC_50_-guided exposure designs have been used in *D. rerio*, where exposure to PS-MPs at concentrations exceeding 100 μg/L for 21 days resulted in particle accumulation in the intestinal region (Qiang et al., 2020). Thus, the concentrations used in the present study should primarily be considered as LC_50_-derived mechanistic hazard-assessment concentrations rather than direct representations of environmental exposure levels. Further studies incorporating field-based concentrations, chemical leachate analysis and mechanistic endpoints are required to establish the environmental significance and underlying mechanisms of PS-MP toxicity.

FTIR analysis qualitatively confirmed the presence of PS-MPs in the digestive tract contents of *D. rerio.* O-H and C-H stretching bands indicate possible interactions between microplastics and biomolecular constituents (Jung et al., 2018). The amide groups are associated with proteins. Alterations in these protein-related bands may reflect interactions between accumulated microplastics and gut tissue proteins, potentially leading to conformational modifications or protein adsorption onto the particle surface. Such interactions have been recognized as an important mechanism influencing microplastic toxicity and bioavailability in aquatic organisms (Wright and Kelly, 2017). The carbonyl and C-O stretching bands indicate surface oxidation or biotransformation of the retained particles (Kappler et al., 2016). All these findings confirm the accumulation and persistence of PS-MPs within the fish gut tissue.

The hematological changes show elevated RBC count observed in PS-MP-exposed fish may represent a compensatory response to oxidative stress and increased oxygen demand, whereas the reduction in WBC count suggests microplastic-induced immunosuppression and disruption of hematopoietic function. Similar hematological alterations have been associated with ROS generation and immune dysregulation following microplastic exposure in fish (Lu et al., 2016; Qiao et al., 2019). Simultaneously, the reduction in plasma glucose and plasma protein levels suggests that PS-MP exposure disrupted energy metabolism and protein homeostasis in fish. Increased energy expenditure during stress responses, coupled with impaired nutrient absorption and hepatic dysfunction, may have contributed to the observed hypoglycemia and hypoproteinemia. Similar alterations have been reported in microplastic-exposed fish and are associated with oxidative stress and metabolic disturbances (Espinosa et al., 2017; Qiao et al., 2019). The observed reductions in plasma glucose and total protein may partly reflect physiological and metabolic responses to PS-MP exposure; however, the contribution of feeding status cannot be completely excluded. Since feeding conditions were maintained consistently among the experimental groups, the observed biochemical alterations are considered primarily in the context of PS-MP exposure.

Several antioxidant and oxidative stress analyses were carried out in gill, gut, and brain tissues treated with PS-MPs. The significant increase in SOD activity in the gill, gut, and brain tissues indicates the activation of the antioxidant defense system in response to PS-MP-induced oxidative stress. Microplastic exposure has been reported to enhance the generation of reactive oxygen species (ROS), which subsequently stimulates antioxidant enzymes such as SOD to protect cells from oxidative damage. As the first line of enzymatic defense, SOD catalyzes the conversion of superoxide radicals into hydrogen peroxide and molecular oxygen, thereby maintaining cellular redox homeostasis (Ding et al., 2018). The elevated SOD activity observed in the gill and gut may be associated with their direct exposure to and accumulation of microplastics, resulting in increased ROS production and oxidative burden. Likewise, the increased SOD activity in brain tissue suggests that PS-MPs can induce neurotoxic effects through oxidative stress pathways, triggering compensatory antioxidant responses. Similar increases in SOD activity have been widely reported in fish exposed to microplastics, confirming oxidative stress as a major mechanism of microplastic-induced toxicity (Devapriya et al., 2026).

In contrast, the significant reduction in GST activity in the gill, gut, and brain tissues indicates impairment of the cellular detoxification system following PS-MP exposure. GST is a key phase II detoxification enzyme that conjugates glutathione with reactive metabolites and xenobiotics, thereby protecting cells against oxidative damage. Excessive production of reactive oxygen species (ROS) induced by microplastic accumulation can overwhelm the antioxidant defense system, leading to depletion of glutathione reserves and subsequent inhibition of GST activity (Subaramaniyam et al., 2023). Furthermore, prolonged oxidative stress may cause structural damage to antioxidant enzymes and disrupt redox homeostasis, resulting in reduced GST activity in target tissues (Patra et al., 2022). Similar declines in GST activity have been reported in fish exposed to microplastics, indicating compromised detoxification capacity and enhanced susceptibility to oxidative injury (Jandu and Vashishat, 2021; Devapriya et al., 2026).

The increased level of GPx activity in the gill, gut, and brain tissues indicates an adaptive response to oxidative stress induced by PS-MPs exposure. GPx plays a vital role in scavenging hydrogen peroxide and lipid hydroperoxides generated during excessive ROS production, thereby protecting cellular membranes from oxidative damage (Li et al., 2022). The elevated GPx activity suggests activation of antioxidant defense mechanisms to maintain cellular redox balance in tissues directly affected by microplastic accumulation and toxicity (Xue et al., 2021). Similar increases in GPx activity have been reported in zebrafish exposed to microplastics and are considered a protective response against ROS-mediated cellular injury (Immaculate et al., 2025).

The elevated LPO level in gill tissue indicates enhanced oxidative damage to membrane lipids resulting from excessive ROS generated during PS-MP exposure. As the primary site of waterborne contaminant contact, the gill is particularly susceptible to oxidative injury, leading to increased lipid peroxidation and cellular membrane disruption (Hamed et al., 2020; Kim et al., 2021). Simultaneously, the significant decrease in AChE activity in the brain tissue indicates an altered neurophysiological response following PS-MP exposure. Inhibition of AChE leads to the accumulation of acetylcholine at neuronal synapses, impairing nerve signal transmission and affecting neurological functions (Barboza et al., 2018). Furthermore, microplastic-induced oxidative stress can damage neuronal cells and alter enzyme activity, thereby reducing AChE levels in brain tissue. Similar reductions in brain AChE activity have been reported in fish exposed to microplastics and are considered a sensitive biomarker of neurotoxic stress (Espinosa et al., 2017). The observed neurotoxic response may arise from direct particle-associated effects or from indirect systemic stress associated with gill injury, oxidative imbalance, and physiological disturbances. Although the PS-MPs exhibited micrometre-scale dimensions by SEM (approximately 1–200 µm), DLS analysis showed a hydrodynamic diameter of 1,115.5 nm with a PDI of 0.569, suggesting the presence of aggregated particles in the aqueous exposure medium. Direct translocation of PS-MPs into the brain or across the blood–brain barrier was not demonstrated in the present study. Therefore, the observed AChE inhibition and brain histopathological alterations cannot be attributed conclusively to direct particle translocation. Further studies using particle-tracking or tissue-specific analytical approaches are required to establish PS-MPs localization in brain tissue and clarify the mechanism of neurotoxicity.

An *a priori* power analysis was not performed before the experiment, and the sample size was based on the experimental design and available biological replicates. Although observed power was calculated for the statistical analyses, the possibility of false-positive findings arising from the evaluation of multiple biochemical, hematological, and tissue-specific endpoints should be considered when interpreting the results. Future studies should incorporate prospective power calculations and appropriate control of multiple testing.

Finally, the histopathological alterations observed in the gills, intestine, and brain of *D. rerio* indicate that PS-MPs induce multi-organ toxicity in a concentration-dependent manner. The degeneration of gill lamellae, branchial arterioles, and chondrocytes suggests impaired respiratory and osmoregulatory functions, consistent with previous reports of microplastic-induced oxidative stress and tissue damage in zebrafish gills (Umamaheswari et al., 2021). Intestinal lesions, including epithelial lifting, vacuolization, and villus shortening, indicate compromised nutrient absorption and intestinal barrier integrity, as reported by Teng et al. (2022). Brain abnormalities such as edema, pyknotic nuclei, necrosis, and granular layer disintegration indicate substantial tissue injury following microplastic exposure. These alterations, together with the observed reduction in brain AChE activity, indicate an altered neurophysiological response to PS-MP exposure (Sökmen et al., 2020; Li et al., 2024). The more pronounced histopathological alterations observed in Treatment II compared with Treatment I are consistent with a concentration-related response to PS-MP exposure. The histopathological assessment in the present study was primarily qualitative. Future investigations incorporating standardized semi-quantitative lesion scoring, such as the protocol proposed by Bernet et al. (1999) would provide a more objective evaluation of lesion severity among treatment groups.

Overall, the present findings demonstrate that exposure to LC_50_-derived sub-lethal concentrations of PS-MPs can induce significant physiological, biochemical, and histopathological disturbances in *D. rerio*. The detection of PS-associated spectral features in the gastrointestinal tract was coupled with alterations in hematological parameters, plasma biochemistry, antioxidant enzymes, lipid peroxidation, and AChE activity. The observed increase in SOD and GPx activities, together with elevated LPO levels and reduced GST and AChE activities, indicates an imbalance between reactive oxygen species generation and antioxidant defense capacity. These responses collectively highlight the susceptibility of gill, gut, and brain tissues to microplastic-induced damage and emphasize the potential ecological risks posed by PS-MPs in freshwater ecosystems. Therefore, continuous environmental monitoring and further investigations on the long-term impacts of PS-MPs are essential for understanding their consequences on aquatic biodiversity and ecosystem health.

## 5 Conclusion

This study demonstrates that PS-MPs induce sub-chronic toxicity in *D. rerio*, as evidenced by hematological alterations, changes in plasma biochemical parameters, oxidative stress responses, altered neurophysiological responses, and histopathological changes in the gill, gut, and brain. The observed increases in SOD, GPx, and LPO, together with reductions in GST and AChE activities, indicate disturbances in antioxidant defense and physiological homeostasis following PS-MP exposure. The concentration-related responses across several biomarkers further demonstrate the adverse effects of PS-MPs under the tested experimental conditions. However, the exposure concentrations were derived from the LC_50_ and should therefore be considered hazard-assessment concentrations rather than direct representations of environmental levels. Further molecular and mechanistic studies are warranted to clarify the pathways underlying PS-MPs toxicity and their potential long-term implications for aquatic organisms.

## Data Availability

The original contributions presented in the study are included in the article/Supplementary Material, further inquiries can be directed to the corresponding author.

## References

[B1] Alhazmi H. Almansour F. H. Aldhafeeri Z. (2021). Plastic waste management: a review of existing life cycle assessment studies. Sustainability 13, 5340. 10.3390/su13105340

[B2] Ansari I. Arora C. Verma A. El Din Mahmoud A. El-Kady M. M. Rajarathinam R. (2025). A critical review on biological impacts, ecotoxicity, and health risks associated with microplastics. Water Air Soil Pollut. 236, 88. 10.1007/s11270-024-07731-z

[B3] APHA (2005). Standard Methods for the Examination of Water and Wastewater. 21st ed. ashington, DC: American Public Health Association.

[B4] Arthur C. Baker J. E. Bamford H. A. (2009). Proceedings of the International Research Workshop on the Occurrence, Effects, and Fate of Microplastic Marine Debris, September 9–11, 2008. Tacoma, WA, USA: University of Washington Tacoma. Available online at: https://repository.library.noaa.gov/view/noaa/2509 (Accessed October 5, 2026).

[B5] Barboza L. G. A. Vieira L. R. Branco V. Figueiredo N. Carvalho F. Carvalho C. (2018). Microplastics cause neurotoxicity, oxidative damage and energy-related changes and interact with the bioaccumulation of mercury in the European seabass, *dicentrarchus labrax* (linnaeus, 1758). Aquat. Toxicol. 195, 49–57. 10.1016/j.aquatox.2017.12.008 29287173

[B6] Bernet D. B. Schmidt H. Meier W. Patricia B. H. Wahli T. (1999). Histopathology in fish: proposal for a protocol to assess aquatic pollution. J. Fish. Dis. 22, 25–34. 10.1046/J.1365-2761.1999.00134.X

[B7] Bliss C. I. (1935). The calculation of the dosage–mortality curve. Ann. Appl. Biol. 22, 134–167. 10.1111/j.1744-7348.1935.tb07713

[B8] Bomski H. (1983). Podstawowe Laboratoryjne Badania Haematologiczne (Basic Laboratory Haematological Analyses). Warszawa: PZWL, 300.

[B9] Borges R. S. Pereira A. C. M. Souza G. C. D. Carvalho J. C. D. (2020). “Histopathology of zebrafish (*Danio rerio*) in nonclinical toxicological studies of new compounds,” in Zebrafish in Biomedical Research, 55. 10.5772/intechopen.88639

[B10] Colovic M. B. Krstic D. Z. Lazarevic-Pasti T. D. Bondzic A. M. Vasic V. M. (2013). Acetylcholinesterase inhibitors: pharmacology and toxicology. Curr. Neuropharmacol. 11, 315–335. 10.2174/1570159X11311030006 24179466 PMC3648782

[B11] Cooper G. R. McDaniel V. Baer D. M. Dubowski K. Morrison D. B. Vanderlinde R. (1970). The determination of glucose by the ortho-toluidine method (filtrate and direct procedure). Stand. Methods Clin. Chem. 6, 159–170. 10.1016/B978-0-12-609106-9.50021-X

[B12] Devapriya P. Malik M. S. Kalpana M. Kalaivani S. Devi V. Raguvaran K. (2026). Biological alterations of polyvinyl chloride (PVC) microplastics on *Danio rerio* at environmentally relevant concentrations – risk assessment. Fish. Physiol. Biochem. 52, 38. 10.1007/s10695-026-01664-6 41774229

[B13] Devasagayam T. P. Tarachand U. (1987). Decreased lipid peroxidation in the rat kidney during gestation. Biochem. Biophys. Res. Commun. 145, 134–138. 10.1016/0006-291X(87)91297-6 3593335

[B14] Ding J. Zhang S. Razanajatovo R. M. Zou H. Zhu W. (2018). Accumulation, tissue distribution, and biochemical effects of polystyrene microplastics in the freshwater fish red tilapia (*Oreochromis niloticus*). Environ. Pollut. 238, 1–9. 10.1016/j.envpol.2018.03.001 29529477

[B15] Efimova I. Bagaeva M. Bagaev A. Kileso A. Chubarenko I. P. (2018). Secondary microplastics generation in the sea swash zone with coarse bottom sediments: laboratory experiments. Front. Mar. Sci. 5, 313. 10.3389/fmars.2018.00313

[B16] Ellman G. L. Courtney K. D. Andres Jr., V. Featherstone R. M. (1961). A new and rapid colorimetric determination of acetylcholinesterase activity. Biochem. Pharmacol. 7, 88–95. 10.1016/0006-2952(61)90145-9 13726518

[B17] Espinosa C. Cuesta A. Esteban M. A. (2017). Effects of dietary polyvinylchloride microparticles on general health, immune status and expression of several genes related to stress in gilthead seabream (*Sparus aurata* L.). Fish. Shellfish Immunol. 68, 251–259. 10.1016/j.fsi.2017.07.006 28684324

[B18] Gao H. Liu C. Wang H. Shen H. (2023). Raman spectra characterization of size-dependent aggregation and dispersion of polystyrene particles in aquatic environments. Chemosphere 333, 138939. 10.1016/j.chemosphere.2023.138939 37182713

[B19] Habig W. H. Pabst M. J. Jakoby W. B. (1974). Glutathione S-transferases: the first enzymatic step in mercapturic acid formation. J. Biol. Chem. 249, 7130–7139. 10.1016/S0021-9258(19)42083-8 4436300

[B20] Hamed M. Soliman H. A. Osman A. G. Sayed A. E. D. H. (2020). Antioxidants and molecular damage in nile tilapia (*Oreochromis niloticus*) after exposure to microplastics. Environ. Sci. Pollut. Res. 27, 14581–14588. 10.1007/s11356-020-07898-y 32048193 PMC7190598

[B21] Han Y. Liu Y. Wang M. Xue Y. (2022). Effects of BPZ, BPC, BPF, and BPS exposure on adult zebrafish (*danio rerio*): accumulation, oxidative stress, and gene expression. Int. J. Environ. Res. Public Health 19, 15784. 10.3390/ijerph192315784 36497860 PMC9739024

[B22] Hayes J. D. Flanagan J. U. Jowsey I. R. (2005). Glutathione transferases. Annu. Rev. Pharmacol. Toxicol. 45, 51–88. 10.1146/annurev.pharmtox.45.120403.095857 15822171

[B23] Horton A. A. (2022). Plastic pollution: when do we know enough? J. Hazard. Mat. 422, 126885. 10.1016/j.jhazmat.2021.126885 34418830

[B24] Howe K. Clark M. D. Torroja C. F. Torrance J. Berthelot C. Muffato M. (2013). The zebrafish reference genome sequence and its relationship to the human genome. Nature 496, 498–503. 10.1038/nature12111 23594743 PMC3703927

[B25] Hwang J. Choi D. Han S. Jung S. Y. Choi J. Hong J. (2020). Potential toxicity of polystyrene microplastic particles. Sci. Rep. 10, 7391. 10.1038/s41598-020-64464-9 32355311 PMC7193629

[B26] Immaculate J. K. Narmatha S. M. Jamila P. (2025). Toxicological assessment of microplastics in zebrafish: biochemical responses and histopathological changes. J. Biochem. Mol. Toxicol. 39, e70518. 10.1002/jbt.70518 40985987

[B27] Iroegbu A. O. C. Ray S. S. Mbarane V. Bordado J. C. Sardinha J. P. (2021). Plastic pollution: a perspective on matters arising: challenges and opportunities. ACS Omega 6, 19343–19355. 10.1021/acsomega.1c02760 34368521 PMC8340096

[B28] Jandu R. S. Vashishat N. (2021). Oxidative stress induced by exposure of microplastics in *Labeo rohita* . Res. Square. 10.21203/rs.3.rs-612018/v1

[B29] Jung M. R. Horgen F. D. Orski S. V. Rodriguez V. Beers K. L. Balazs G. H. (2018). Validation of ATR FT-IR to identify polymers of plastic marine debris, including those ingested by marine organisms. Mar. Pollut. Bull. 127, 704–716. 10.1016/j.marpolbul.2017.12.061 29475714 PMC13077791

[B30] Kappler A. Fischer D. Oberbeckmann S. Schernewski G. Labrenz M. Eichhorn K. J. (2016). Analysis of environmental microplastics by vibrational microspectroscopy: FTIR, raman or both? Anal. Bioanal. Chem. 408, 8377–8391. 10.1007/s00216-016-9956-3 27722940

[B31] Kim J. H. Yu Y. B. Choi J. H. (2021). Toxic effects on bioaccumulation, hematological parameters, oxidative stress, immune responses and neurotoxicity in fish exposed to microplastics: a review. J. Hazard. Mat. 413, 125423. 10.1016/j.jhazmat.2021.125423 33930961

[B32] Kooi M. Primpke S. Mintenig S. M. Lorenz C. Gerdts G. Koelmans A. A. (2021). Characterizing the multidimensionality of microplastics across environmental compartments. Water Res. 202, 117429. 10.1016/j.watres.2021.117429 34304075

[B33] Kostyuk V. A. Potapovich A. I. (1989). Superoxide-driven oxidation of quercetin and a simple sensitive assay for determination of superoxide dismutase. Biochem. Int. 19, 1117–1124. 2561443

[B34] Lei L. Wu S. Lu S. Liu M. Song Y. Fu Z. (2018). Microplastic particles cause intestinal damage and other adverse effects in zebrafish *Danio rerio* and nematode *Caenorhabditis elegans* . Sci. Total Environ. 619–620, 1–8. 10.1016/j.scitotenv.2017.11.103 29136530

[B35] Li Z. Chang X. Hu M. Fang J. K. Sokolova I. M. Huang W. (2022). Is microplastic an oxidative stressor? Evidence from a meta-analysis on bivalves. J. Hazard. Mat. 423, 127211. 10.1016/j.jhazmat.2021.127211 34844346

[B36] Li X. Zheng T. Zhang J. Chen H. Xiang C. Sun Y. (2024). Photoaged polystyrene microplastics result in neurotoxicity associated with neurotransmission and neurodevelopment in zebrafish larvae (*Danio rerio*). Environ. Res. 250, 118524. 10.1016/j.envres.2024.118524 38401682

[B60] Leary S. Underwood W. Anthony R. Cartner S. Grandin T. Greenacre C. (2020). AVMA Guidelines for the Euthanasia of Animals: 2020 Edition. Schaumburg, IL, USA: American Veterinary Medical Association. Available online at: https://www.avma.org/sites/default/files/2020-02/Guidelines-on-Euthanasia-2020.pdf.

[B37] Lowry O. H. Rosebrough N. J. Farr A. L. Randall R. J. (1951). Protein measurement with the folin phenol reagent. J. Biol. Chem. 193, 265–275. 10.1016/S0021-9258(19)52451-6 14907713

[B38] Lu Y. Zhang Y. Deng Y. Jiang W. Zhao Y. Geng J. (2016). Uptake and accumulation of polystyrene microplastics in zebrafish (*Danio rerio*) and toxic effects in liver. Environ. Sci. Technol. 50, 4054–4060. 10.1021/acs.est.6b00183 26950772

[B39] Mohandas J. Marshall J. J. Duggin G. G. Horvath J. S. Tiller D. J. (1984). Differential distribution of glutathione and glutathione-related enzymes in rabbit kidney: possible implications in analgesic nephropathy. Biochem. Pharmacol. 33, 1801–1807. 10.1016/0006-2952(84)90353-8 6145422

[B40] Napper I. E. Thompson R. C. (2023). Plastics and the environment. Annu. Rev. Environ. Resour. 48, 55–79. 10.1146/annurev-environ-112522-072642

[B41] Nugnes R. Lavorgna M. Orlo E. Russo C. Isidori M. (2022). Toxic impact of polystyrene microplastic particles in freshwater organisms. Chemosphere 299, 134373. 10.1016/j.chemosphere.2022.134373 35314178 PMC8932136

[B42] Patra I. Huy D. T. N. Alsaikhan F. Opulencia M. J. C. Tuan P. V. Nurmatovaet K. C. (2022). Toxic effects on enzymatic activity, gene expression and histopathological biomarkers in organisms exposed to microplastics and nanoplastics: a review. Environ. Sci. Eur. 34, 80. 10.1186/s12302-022-00652-w

[B43] Qiang L. Lo L. S. H. Gao Y. Cheng J. (2020). Parental exposure to polystyrene microplastics at environmentally relevant concentrations has negligible transgenerational effects on zebrafish (*Danio* rerio). Ecotoxicol. Environ. Saf. 206, 111382. 10.1016/j.ecoenv.2020.111382 33011509

[B44] Qiao R. Lu K. Deng Y. Ren H. Zhang Y. (2019). Combined effects of polystyrene microplastics and natural organic matter on the accumulation and toxicity of copper in zebrafish. Sci. Total Environ. 682, 128–137. 10.1016/j.scitotenv.2019.05.163 31117014

[B45] Rochman C. M. Brookson C. Bikker J. Djuric N. Earn A. Bucci K. (2019). Rethinking microplastics as a diverse contaminant suite. Environ. Toxicol. Chem. 38, 703–711. 10.1002/etc.4371 30909321

[B46] Rusia V. Sood S. K. (1992). Routine haematological tests. Med. Lab. Technol. 1, 252–258.

[B47] Siddiqui S. A. Singh S. Bahmid N. A. Shyu D. J. Domínguez R. Lorenzo J. M. (2023). Polystyrene microplastic particles in the food chain: characteristics and toxicity. A review. Sci. Total Environ. 886, 164531. 10.1016/j.scitotenv.2023.164531 37268142

[B48] Sokal R. R. Rohlf J. F. (2013). Biometry: The Principles and Practice of Statistics in Biological Research. 4th ed. New York: W.H. Freeman and Company, 157–162. Available online at: https://searchworks.stanford.edu/view/9754871 (Accessed October 5, 2026).

[B49] Sökmen T. Ö. Sulukan E. Türkoğlu M. Baran A. Özkaraca M. Ceyhun S. B. (2020). Polystyrene nanoplastics (20 nm) are able to bioaccumulate and cause oxidative DNA damages in the brain tissue of zebrafish embryo (*Danio rerio*). Neurotoxicology 77, 51–59. 10.1016/j.neuro.2019.12.010 31862285

[B50] Subaramaniyam U. Allimuthu R. S. Vappu S. Ramalingam D. Balan R. Paital B. (2023). Effects of microplastics, pesticides and nano-materials on fish health, oxidative stress and antioxidant defense mechanism. Front. Physiol. 14, 1217666. 10.3389/fphys.2023.1217666 37435307 PMC10331820

[B51] Teng M. Zhao X. Wang C. Wang C. White J. C. Zhao W. (2022). Polystyrene nanoplastics toxicity to zebrafish: dysregulation of the brain–intestine–microbiota axis. ACS Nano 16, 8190–8204. 10.1021/acsnano.2c01872 35507640

[B52] Umamaheswari S. Priyadarshinee S. Kadirvelu K. Ramesh M. (2021). Polystyrene microplastics induce apoptosis via ROS-mediated p53 signaling pathway in zebrafish. Chem. Biol. Interact. 345, 109550. 10.1016/j.cbi.2021.109550 34126101

[B53] Vinodh R. Abidov A. Peng M. M. Babu C. M. Palanichamy M. Cha W. S. (2015). A new strategy to synthesize hypercross-linked conjugated polystyrene and its application towards CO_2_ sorption. Fibers Polym. 16, 1458–1467. 10.1007/s12221-015-5151-y

[B54] Wiedner K. Polifka S. (2020). Effects of microplastic and microglass particles on soil microbial community structure in an arable soil (chernozem). Soil 6, 315–324. 10.5194/soil-6-315-2020

[B55] Witeska M. Kondera E. Ługowska K. Bojarski B. (2022). Haematological methods in fish–not only for beginners. Aquaculture 547, 737498. 10.1016/j.aquaculture.2021.737498

[B56] Wright S. L. Kelly F. J. (2017). Plastic and human health: a micro issue? Environ. Sci. Technol. 51, 6634–6647. 10.1021/acs.est.7b00423 28531345

[B57] Xue Y. H. Feng L. S. Xu Z. Y. Zhao F. Y. Wen X. L. Jin T. (2021). The time-dependent variations of zebrafish intestine and gill after polyethylene microplastics exposure. Ecotoxicology 30, 1997–2010. 10.1007/s10646-021-02469-4 34529203

[B58] Yang H. Zhu Z. Xie Y. Zheng C. Zhou Z. Zhu T. (2022). Comparison of the combined toxicity of polystyrene microplastics and different concentrations of cadmium in zebrafish. Aquat. Toxicol. 250, 106259. 10.1016/j.aquatox.2022.106259 35932501

[B59] Yu Q. Hu X. Yang B. Zhang G. Wang J. Ling W. (2020). Distribution, abundance and risks of microplastics in the environment. Chemosphere 249, 126059. 10.1016/j.chemosphere.2020.126059 32062205

